# Invivo biocompatibility determination of acellular aortic matrix of buffalo origin

**DOI:** 10.1007/s40204-014-0027-6

**Published:** 2014-09-23

**Authors:** Devarathnam Jetty, Ashok Kumar Sharma, Naveen Kumar, Sameer Shrivastava, B. Sonal, R. B. Rai

**Affiliations:** 1 Division of Surgery, Indian Veterinary Research Institute, Izatnagar, 243122 Uttar Pradesh India; 2 Division of Veterinary Biotechnology, Indian Veterinary Research Institute, Izatnagar, 243122 Uttar Pradesh India; 3 Division of Pathology, Indian Veterinary Research Institute, Izatnagar, 243122 Uttar Pradesh India

**Keywords:** Acellular matrix, Acellular aortic matrix, Buffalo aortic matrix, BDDGE, EDC

## Abstract

In the present study, biocompatibility of native, acellular, 1,4-butanediol diglycidylether and 1-ethyl-3-(3-dimethyl aminopropyl carbodiimide (EDC) cross-linked acellular aortic grafts was evaluated following subcutaneous implantation in guinea pigs. Biocompatibility was evaluated based on macroscopic, histopathological observations and immune responses elicited by the implanted grafts. Results showed that macroscopically, no abnormal cellular reaction was observed at the host–graft junction in any of the implanted animals. Histopathological observations revealed that the inflammatory response was mild during first 15 days post-implantation and increased at 30 days post-implantation in acellular and cross-linked tissues. By day 60, marked ingrowth of host tissue was observed in EDC cross-linked acellular aortic grafts. ELISA and lymphocyte proliferation assay revealed that animals implanted with EDC grafts showed least immune response when compared to others. Therefore, it was concluded that EDC cross-linked acellular aortic grafts were more compatible and had better handling qualities than the other cross-linked grafts.

## Introduction

Acellular matrices offer a new approach in the management of abdominal wall defects because of their potential capacity to resist infection and induce a milder inflammatory response, angiogenesis and host cell migration. The structural characteristics and mechanical properties of acellular matrices are dependent upon tissue from which they are harvested (Badylak 2004). Extraction of cellular components from tissue, minimize immunologically induced inflammatory process (Wilson et al. 1995). Various acellular materials from different tissue sources are being used in abdominal wall reconstructions which are even available commercially. But the complications (Nahabedian 2007) associated with these materials led to the search of alternate sources. The blood vessel matrix derived from porcine aorta served as a viable option in the repair of abdominal wall tissue defects (Bellows et al. 2008). But there is limitation in getting large size scaffolds due to narrow width and small area of porcine aortic matrix which can be used for the repair of large size abdominal wall defects in bovines and equines. Therefore, xenogenic vascular matrix of buffalo origin was considered as an alternative to porcine source. Cross-linking improves mechanical properties and enhances resistance to degradation (Schmidt and Baier 2000). Before biomaterials can be applied for its clinical use, the tissue response to these biomaterials had to be evaluated in vivo. This approach is to identify a suitable xenogenic tissue and modify the structure to give a material that will be immunologically inert, mechanically robust, and will support cell attachment and proliferation (Schmidt and Baier 2000). Cross-linking may prove effective for lowering immunogenicity by altering the display of antigenic determinants (Yannas 1996). Acellular aortic grafts cross-linked with 1 % 1-ethyl-3-(3-dimethyl aminopropyl carbodiimide (EDC) and 1 % 1,4-butanediol diglycidylether (BDDGE) for 24 h showed promising results during in vitro studies (Devarathnam et al. 2014). In this context, acellular aortic tissue of buffalo origin cross-linked with BDDGE and EDC was evaluated in vivo for its efficacy in abdominal wall reconstruction. In the present study biocompatibility of native, acellular, BDDGE and EDC cross-linked acellular aortic grafts was evaluated following subcutaneous implantation in guinea pigs.

## Materials and methods

### Decellularization

Fresh posterior abdominal aorta of buffalo origin was collected from the local abattoir and immediately preserved in ice-cold sterile phosphate buffered saline (pH 7.4) containing 1 % sodium azide (Merck, limited, Mumbai) and 0.02 % EDTA (Merck limited, Mumbai). The maximum time period between tissue procurement and processing was <4 h. The extraneous fat and fascia were carefully removed and the aorta was cut into 2 × 2 cm^2^ pieces for decellularization. Each aortic tissue sample was treated with 20 ml of 1 % sodium dodecyl sulphate (SDS) (SD fine chem. limited, Mumbai) solution for 48 h at 37 °C with continuous shaking in an orbital shaker at the rate of 180 rpm. Samples were then thoroughly washed with 1 % phosphate buffered saline solution.

### Cross-linking

The acellular tissues obtained after decellularization were fixed in 1 % BDDGE (Sigma-Aldrich, USA) and 1 % EDC (Sisco Research laboratory, Mumbai) at 37 °C for 24 h. The aqueous solutions of BDDGE and EDC were buffered with phosphate buffered saline (PBS). The amount of solution used to cross-link each sample was 20 ml. The cross-linked aortic tissues were thoroughly washed with PBS by changing the solution several times and were prepared for subcutaneous study.

### In vivo study

**Fig. 1:**
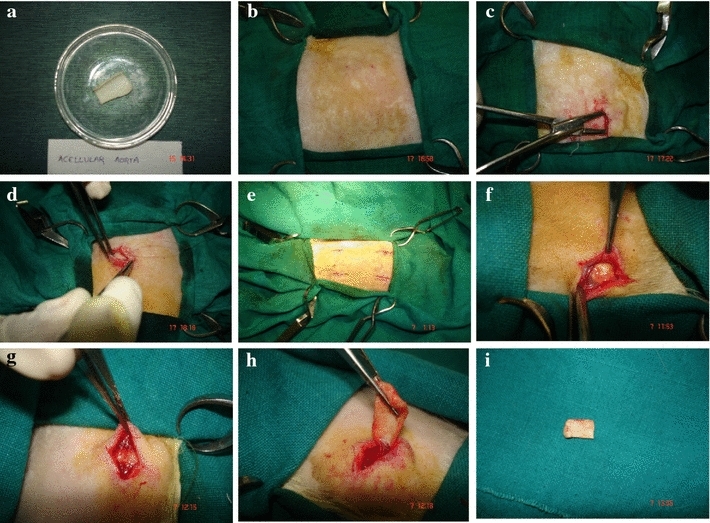
Subcutaneous implantation (**a**–**f**) and retrieval (**g**–**i**) of aortic matrix grafts in a guinea pig model

Native, acellular and cross-linked acellular aortic grafts of 20 × 10 mm size were implanted subcutaneously on either side of the spine in 16 albino guinea pigs which were randomly divided into four groups. Animals were anaesthetized using xylazine and ketamine anaesthetic combination. The animals were restrained in sternal recumbency. Dorsal thoracic area was prepared for aseptic surgery. On either side of the spine two 1-cm-long skin incisions were made at a distance of 5 cm apart and 2 cm lateral to the spine on both left and right side and subcutaneous pouches were created. The implants were pushed in the pockets and were anchored to subcutaneous tissue using polyamide suture no 1-0. The skin incision was closed with simple interrupted sutures using same suture material. The native, acellular and cross-linked aortic tissues were implanted in separate guinea pigs. These grafts were retrieved back on 15, 30, and 60 post-operative implantation days and subjected to following observations (Fig. 1).

#### Macroscopic observations

Macroscopic assessment of the retrieved implant was done as per the procedure described by Lu et al. (1998).

#### Microscopic observations

The retrieved implants were preserved in 10 % formalin saline solution. The tissues were processed by routine paraffin embedding technique and the sections were cut at 5 µm thickness. The sections were stained with hematoxylin and eosin (H&E) by using the method described by Tyrell et al. (1989) to evaluate the tissue reaction. The sections were examined for inflammatory reaction around the implant material, degenerative changes of the graft, neovascularization, lymphocytes infiltration and fibroblastic proliferation.

#### Immunological studies

Lymphocyte proliferation assay and ELISA were undertaken to evaluate the immune responses of subcutaneously implanted cross-linked aortic tissues.

##### Lymphocyte proliferation assay

The cell-mediated immune response toward xenogenic acellular aortic matrix graft was studied by lymphocyte proliferation assay

*Preparation of antigen:* The cross-linked acellular aortic matrix grafts were cut into small pieces and extract was made by grinding with sterile glass powder in sterile normal saline solution containing penicillin and streptomycin at a concentration of 100 IU/ml and 1 µg/ml, respectively. The samples were centrifuged at 2,000 rpm for 30 min and supernatant was filtered through 0.22-µm syringe filter and used in the assay to stimulate lymphocytes in vitro. The uncross-linked acellular and native aortic tissues were also processed similarly and used in the assay to stimulate T cells so as to compare the stimulation index with cross-linked graft.

*Procedure:* Blood (2 ml) was aseptically collected from guinea pig from anterior vena cava in heparinized tubes on 0, 15 and 60 post-implantation days. Sterile PBS (2 ml) was added to the 2 ml of blood and properly mixed. It was layered carefully over 2 ml of lymphocyte separation medium (Histopaque 1077, Sigma Aldrich Co., St. Louis) and centrifuged at 2,200 rpm for 30 min. The buffy coat was collected in a fresh tube and two washings were done with sterile PBS at 1,800 rpm for 10 min. Supernatant was discarded and pellet was resuspended in RPMI 1640 growth medium (Sigma Aldrich, USA). The cells were adjusted to a concentration of 2 × 10^6^ viable cells/ml in RPMI 1640 growth medium and seeded in 96-well tissue culture plate @ 100 µl/well. The cells were incubated at 37 °C in 5 % CO_2_ environment. Cells from each guinea pig were stimulated with antigen (10–20 μg/ml) and PHA (10 μg/ml) in triplicates and three wells were left unstimulated for each sample.

After 45 h, 40 µl of MTT solution (5 mg/ml) was added to all the wells and incubated further for 4 h. The plates were then centrifuged for 15 min in plate centrifuge at 2,500 rpm. The supernatant was discarded, plates dried and 150 µl DMSO was added to each well and mixed thoroughly by repeated pipetting to dissolve the formazan crystals. The plates were immediately read at 570 nm with 620 nm as reference wavelength. The stimulation index (SI) was calculated using the following formula:

$$\text{Stimulation index (SI)}=\frac{\text{OD of stimulated cultures}}{\text{OD of unstimulated cultures}}.$$

##### ELISA

To evaluate the immunocompatibility of the cross-linked biomaterials ELISA was performed. Serum samples from the guinea pigs were collected on 15, 30, and 60 post-implantation days for ELISA. The test was done as per standard protocol. Microtitre ELISA plate (Nunc, Denmark) was coated with 0.25 μg of protein (derived from grafted material) in 100 μl of 0.05 M sodium carbonate buffer (pH 9.6) per well. The plate was incubated at 4 °C overnight. After incubation plate was washed with PBS-T (0.15 M sodium chloride 0.02 M phosphate buffer (pH 7.2) containing 0.005 % Tween 20). Subsequently, blocking was done with 1 % bovine serum albumin in PBS-T and further incubated at 37 °C for 2 h. Plate was washed with PBS-T followed by adding 1:100 dilution of sera obtained from different guinea pigs grafted with various graft materials. The plate was incubated again for 2 h at 37 °C, followed by washing with PBS-T. Peroxidase-labelled anti-guinea pig conjugate 1:20,000 dilutions was made in PBS-T and instilled 100 μl in each well and then incubated at 37 °C for 2 h. Finally plate was washed as before and peroxidase substrate was added [100 μl of 17 mM Na citrate buffer, pH 6.3 containing 0.2 % (wt/vol.) *O*-phenylene diamine and 0.015 % (wt/vol.) hydrogen peroxide] per well. Substrate was allowed to act for 30 min at 37 °C, keeping the plate in dark. Absorbance was recorded at 492 nm using ELISA reader (ECIL, Hyderabad). The values of antibodies titre (absorbance) were expressed in ng/ml.

### Statistical analysis

The data were analysed by ANOVA and Student’s *t* test as per Snedecor and Cochran (1973). The statistical analysis was done using statistical soft ware (SPSS vr 14)

## Results

### Macroscopic observations

All the aortic grafts were covered by white fibrous connective tissue which was thin initially and became dense as the days progressed. By day 60, the implanted biomaterials were more deeply seated within the fibrous connective tissue and were difficult to retrieve. No change in colour and consistency was observed in native, acellular and EDC cross-linked aortic grafts. Complications like infection or pus formation was not seen in the vicinity of any of the implanted biomaterials.

### Microscopic observations

**Fig. 2:**
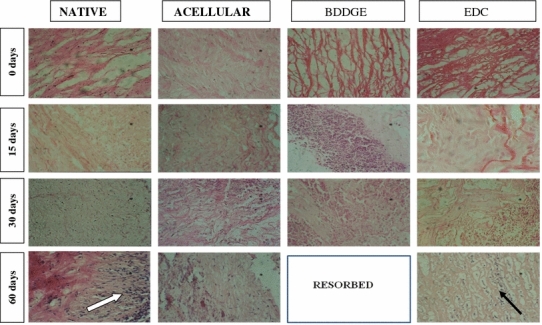
Photomicrographs of native, acellular, BDDGE and EDC cross-linked acellular aortic grafts retrieved at 15, 30 and 60 days after subcutaneous implantation in guinea pig model (H&E stain, ×40). Native grafts showing chronic inflammatory response (*white arrow*) at day 60. BDDGE cross-linked grafts got resorbed by day 60. EDC cross-linked grafts showing development of connective tissue with mature fibroblasts (*black arrow*) at day 60

On day 15, native aortic matrix showed extensive infiltration of mononuclear cells comprising macrophages and epithelioid cells indicating chronic inflammatory response. The collagen fibres were moderately degraded. Formation of a delimiting membrane around the layer of cellular infiltration was also observed. However, by day 30, the inflammatory reaction was remarkably reduced and there was proliferation of fibrous tissue on the outer layer. On day 60, the graft was surrounded on one side by proliferating connective tissue, with infiltration of mononuclear cells and fibroblasts indicating chronic inflammatory response (Fig. 2). There was marked ingrowth of host tissue in the graft with infiltration of mononuclear cells and fibroblasts at different stages of maturation.

On day 15, the acellular aortic matrix showed mild chronic inflammatory response with less infiltration of mononuclear cells when compared to the native aortic matrix graft. Cellular infiltration was limited only to the periphery of graft. Degradation of collagen fibres was mild and confined to the periphery. On day 30, severe inflammatory response was observed at both the interfaces. Moderate degradation of collagen fibres was observed. There was extensive proliferation of fibrous cellular tissue. On day 60, the graft was degraded and covered with connective tissue with fibroblasts at different stages of maturation (Fig. 2).

On day 15, there was severe mononuclear cell infiltration, indicating chronic inflammatory response which was mostly confined to the periphery of the graft. Degradation of collagen fibres was observed only at the surface. On day 30, the cellular infiltration was observed inside the graft. But the inflammatory response was reduced when compared to day 15. Moderate degradation of collagen fibres was observed owing to infiltrating mononuclear cells. On day 60, the graft was resorbed (Fig. 2).

On day 15, there was mild chronic inflammatory response, confined to the periphery of the graft. The interface was covered with thin band of connective tissue. On day 30, the inflammatory response was severe with mild to moderate degradation of collagen fibres. The graft was enveloped by thick fibrous tissue reaction. On day 60, there was development of connective tissue with matured fibroblasts (Fig. 2).

### Immunological studies

#### Lymphocyte proliferation assay

**Fig. 3:**
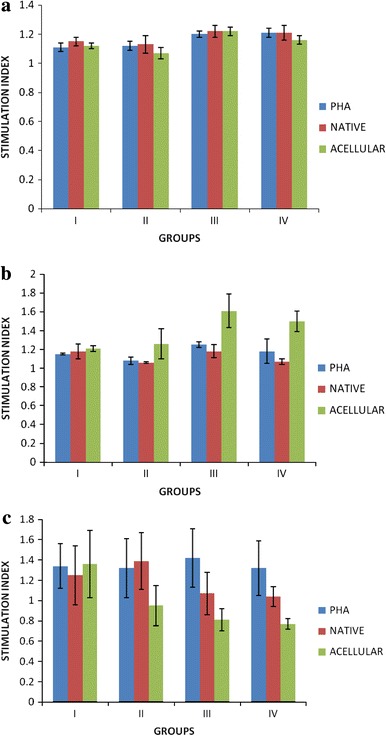
**a** Mean ± SE of stimulation index (SI) of guinea pigs (peripheral blood lymphocytes) subcutaneously implanted with native (*I*), acellular (*II*), BDDGE (*III*) and EDC (*IV*) cross-linked aortic grafts at day 0, **b** at day 15, **c** at day 60

The cell-mediated immune response towards the subcutaneously implanted native, acellular and cross-linked acellular aortic matrix grafts in all the guinea pigs was assessed by MTT colorimetric assay. The mean ± SE of stimulation index (SI) values of native, acellular and cross-linked acellular aortic matrix grafts at 0, 15 and 60 days post-implantation, stimulated with PHA, native and acellular aortic antigens are presented in Fig. 3a–c. The stimulation index values were lower in group implanted with EDC cross-linked grafts at 15 and 60 days post-implantation when stimulated with both acellular and native antigens.

#### ELISA

**Fig. 4:**
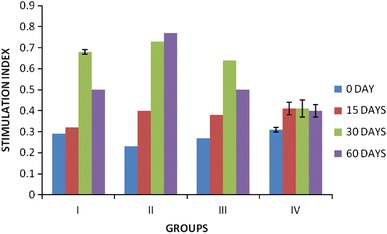
Mean ± SE of absorbance values (ELISA) of guinea pigs subcutaneously implanted with native (*I*), acellular (*II*), BDDGE (*III*) and EDC (*IV*) cross-linked aortic grafts

The humoral immune response elicited by the subcutaneously implanted native, acellular and cross-linked acellular aortic matrix grafts was determined by using indirect ELISA. The serum samples collected on 15, 30 and 60 days post-implantation were evaluated for the levels of antibody generated towards the aortic matrix graft. The anti-graft antibodies were expressed as mean ± SE absorbance at 492 nm wavelength (OD_492_) and are presented in Fig. 4. The levels of antibodies present in serum samples collected prior to implantation were taken as basal values. Hyperimmune sera raised against native aorta were used as standard positive control (1.5 ± 0.18). The anti-graft antibody levels started increasing on 15th post-implantation day in all the groups. The anti-graft antibody levels showed an increasing trend till 30th post-implantation day and then onwards showed a decreasing trend in all groups except acellular graft implanted group, which showed increasing trend up to 60th day post-implantation. Among all the groups, group implanted with EDC cross-linked grafts showed minimal anti-graft antibody levels when compared to native, acellular and BDDGE groups and the levels remained constant and more or less equal to basal value (0.306 ± 0.01).

## Discussion

### Macroscopic observations

In this study, a uniform layer of white connective tissue was found covering all the implanted biomaterials at days 15 and 30 post-implantation. However, it was dense at day 30. Shoukry et al. (1997) also observed similar observations where commercial polyester fabric was used to repair the abdominal hernias in horse. At day 60 the implanted biomaterials were present beneath the fibrous connective tissue. Cross-linking with GA induced cross-links in lysyl amino acid residues of adjacent collagen monomers. This structural change reduces immunogenicity by neutralizing antigenic epitopes, reduces the rate of in vivo degradation. However, it causes significant changes in the mechanical properties such as reduced stress relaxation and increased extensibility. GA-treated tissues are prone to calcification and subjected to fibrous encapsulation following implantation (Nimni et al. 1987). An additional detrimental side effect of GA treatment has been the tendency of such treated tissues to release into the surrounding environment cytotoxic by products such as monomeric GA and hemiacetals and these products caused persistent low-grade local tissue inflammation at the site and cell growth on GA cross-linked materials is markedly diminished (Gendler et al. 1984). On the other hand, the main advantage of using carbodiimides reagents that they induce cross-links between carboxylic acid and amine groups without itself was being incorporated. EDC cross-linking involves activation of the carboxylic acid groups of Asp or Glu residues of EDC to give *O*-acylisourea groups. EDC cross-linking yielding so-called zero length cross-linking because it is not incorporated in the matrix (Lee et al. 1996).

### Microscopic observations

The native aorta induced severe host inflammatory reaction as compared to acellular aorta, characterized by infiltration of mononuclear cells and fibroblasts which persisted up to 60 days post-implantation. It appears, however, that acellular materials that are resistant to degradation elicit a pro-inflammatory type of response, whereas the anti-inflammatory macrophage phenotype predominates with native tissues that are readily degraded.

Acellular aortic matrix showed less host inflammatory reaction as compared to the native tissue for the first 15 days post-implantation suggesting the decreased antigenicity of these matrices due to decellularization. Similar results were obtained using the acellular bovine pericardium by Gilberto and Pereira (2003). Thirty days after implantation, it was found that inflammatory cells and fibroblasts were able to infiltrate into acellular tissues. Penetration of cells into the acellular tissue may be caused by the extraction of soluble proteins, lipids, nucleic acids, salts, and carbohydrates, rendering the tissue more permeable to cellular infiltrates.

The depth of cell infiltration into the acellular tissue decreased with increase in cross-linking degree. In BDDGE and EDC cross-linked grafts, host inflammatory reaction was limited to the surface of the graft. This observation may be attributed to the fact that cross-linking within the acellular tissue may produce a physical barrier for cell infiltration. Additionally, cross-linking of the acellular tissue increased its resistance against enzymatic attack, which is necessary for cell migration into scaffolds. Infiltration of inflammatory cells was accompanied by degradation of collagen fibres. Among the various inflammatory cells such as polymorphonuclear leukocytes, macrophages and fibroblasts that infiltrate implanted materials, macrophages are known to be able to secrete collagenase among other proteases (Silver et al. 1988). This allows the fibroblasts from the host tissue to migrate into implanted grafts. In the present study also, acellular graft was covered with connective tissue with fibroblasts at day 60. In BDDGE cross-linked grafts, moderate degradation of collagen fibres were observed from day 30 post-implantation. By day 60, the grafts were completely absorbed suggesting decreased resistance of graft towards enzymatic attack in vivo. In the present study, EDC cross-linked grafts showed less inflammatory response when compared to native and acellular grafts. Host reaction was limited to the periphery of the graft. EDC increases collagen biostability and reduces antigenicity while preserving compatibility (Hardin-Young et al. 1996). Moreover, by day 60, there was proliferating connective tissue with fibroblasts at end stage of maturation suggesting host ingrowth into the graft.

### Immunological studies

#### Lymphocyte proliferation assay

Lymphocyte proliferation assay (LPA) measures the ability of lymphocytes placed in short-term tissue culture to undergo a clonal proliferation when stimulated in vitro by a foreign molecule (antigen/mitogen). CD4^+^ lymphocytes proliferate in response to antigenic peptides in association with class II major histocompatibility complex (MHC) molecules on antigen-presenting cells (APCs). Graft rejection is usually mediated by activity of T cells, especially cytotoxic T cells. The T cell subsets (Th1 and Th2) generated by naïve T cell on MHC antigen stimulation, play a major role in the graft rejection through activity of different sets of cytokines that activate macrophages and B cells. Cells in extracellular matrices have Class I and II histocompatibility antigens capable of eliciting rejection reactions. Also, the cells have glycoproteins recognized by the immune system of hosts, which elicit rejection reactions. Therefore, if these substances are eliminated from extracellular matrices, rejection reactions can be prevented. Removal of antigens found on cell surface proteins will reduce in vivo cellular attack and possibly eliminate the need for extensive cross-linking (Courtman et al. 1994). However, complete elimination of all antigens is considerably difficult to perform and verify (Malone et al. 1984). In the present study, the antigen prepared from acellular tissue showed highest SI in MTT assay. The SI recorded for cross-linked samples was lower in comparison to the values of uncross-linked samples. The greater ability of this antigen to stimulate the lymphocytes in vitro may be attributed to the fact that on treatment with biological detergent, the bonds between protein molecules are broken and results into a change from quaternary and tertiary structure to primary and secondary structures. Therefore, the acellular antigen had greater ability to trigger CMI response in host because of presence of shorter peptide fragments which can be presented to the immune system by MHC class II pathway and stimulate the CD4^+^ lymphocytes; whereas, the cross-linked tissue is not processed in the body to form shorter immunogenic fragments which can elicit the CMI in host. This may also be because of the fact that on cross-linking tissues with different chemicals, the site where biological enzymes act in vivo, are masked and the cross-linked tissue is no longer broken down into smaller peptide fragments to elicit immune response. Cross-linking of proteins on treatment with EDC might have masked immunogenic epitopes causing delayed immune response (CMI) in the host body. Similar results were reported by Dewangan et al. (2012) with bladder acellular matrix grafts. Cross-linking may prove effective for lowering immunogenicity by altering the display of antigenic determinants (Yannas 1996).

#### ELISA

The presence of antibodies to xenogenic collagen was an epiphenomenon and not an indicator for rejection of the implant (Ruszezak 2003). In the present study, ELISA was performed to check the extent of antibody generated towards the graft components. The absorbance values were taken as a measure to compare the magnitude of immune response. Seddon et al. (2004) reported that ionic detergents like 1 % SDS are effective for solubilizing both cytoplasmic and nuclear cellular membranes, but tend to denature proteins by disrupting protein–protein interactions. Collagens are weakly immunogenic as compared to other proteins. The major antigenic determinants are situated in the telopeptide regions of the molecule. The other two types of determinants are composed of the triple helix and of the amino acid sequence of the alpha chains. The latter type is accessible only when the collagen is denatured (Chevallay and Herbage 2000). When the antigenic determinants are exposed due to collagen degeneration, it results in severe immune response. In the present study, acellular group showed higher immune response when compared to cross-linked and native groups. The antigenicity of a collagen biomaterial can be reduced by the process of cross-linking (O’Brien et al. 1984). Therefore, the immune response was less in BDDGE and EDC cross-linked groups when compared to acellular group. It is a well-known fact that production of antibodies requires about 21 days after antigen administration. As a result, the antibody levels increased in all groups at 30 days post-implantation. As the EDC resulted in efficient masking of antigenic determinant sites, low levels of antibodies were detected at 15, 30 and 60 days post-implantation in the group that received EDC implants.

## Conclusion

In the present study, EDC cross-linked acellular aortic grafts were found more biocompatible when compared to other grafts owing to their least inflammatory and immune responses in in vivo studies.

## Abbreviations

BDDGE: 1,4-Butanediol diglycidylether
EDTA: Ethylene diamine tetra acetic acid
ELISA: Enzyme linked immunosorbent assay
EDC: 1-Ethyl-3-(3-dimethylaminopropyl)carbodiimide
H&E: Hematoxylin and eosin
LPA: Lymphocyte proliferation assay
MHC: Major histocompatibility complex
MNCs: Mononuclear cells
MTT: Methylthiazolyl tetrazolium
PBS: Phosphate buffered saline
PHA: Phytohaemagglutinin
Rpm: Rotations per minute
RPMI: Roswell Park Memorial Institute
SI: Stimulation index
PBS-T: Phosphate buffered saline with Tween-20
ANOVA: Analysis of variance
CD_4_: Cluster of differentiation-4
CMI: Cell-mediated immunity
